# Dataset on nurses' perception and practice of inter-professional collaboration at Muhammadiyah hospitals, Indonesia

**DOI:** 10.1016/j.dib.2020.105863

**Published:** 2020-06-16

**Authors:** Musrifatul Uliyah, Luthfiyah Nurlaela, Mustaji, Abdul Aziz Alimul Hidayat

**Affiliations:** a Departement of Nursing, University Muhammadiyah of Surabaya, 60113, Indonesia; b Universitas Negeri Surabaya, Indonesia; c Universitas Negeri Surabaya, Indonesia; d Departement of Nursing, University Muhammadiyah of Surabaya, 60113, Indonesia

**Keywords:** Nursing, Perception, Interprofessional, Collaboration

## Abstract

This article focused on presenting data collection of nurses' perceptions and practices of interprofessional collaboration at Muhammadiyah hospitals in Six regions in East Java, (Surabaya, Gersik, Lamongan, Sidoarjo, Banyuwangi, and Bojonegoro) Indonesia. The survey was conducted on nurses’ perceptions and practices towards interprofessional education in hospitals. The survey was conducted using a structured questionnaire administered to 312 nurses at Muhammadiyah hospitals in East Java province which was the second largest population after West Java province and the province with the highest number of Muhammadiyah hospitals in Indonesia. The survey involved nurses working at these hospitals and was conducted from June to December 2019. The questionnaire was used for data collection consisted of 24 questions on perception of inter-professional collaboration, and 21 questions on inter-professional collaborative practices using a Likert scale measure. The data were analyzed using quantitative descriptive statistical analysis.

Specifications table

**Table:** 

Subject	Nursing and Health Professions
Specific subject area	Interprofessional collaboration
Type of data	Table
How data were acquired	Field survey, face to face interview.
Data format	Raw data
Parameters for data collection	Nurses who worked in inpatient rooms, those with a minimum of one year work experience
Description of data collection	Data were obtained through a questionnaire administrated to nurses working with a minimum of one year work experience in Muhammadiyah hospitals in East Java, Indonesia.
Data source location in	Six regions (Surabaya, Gersik, Lamongan, Sidoarjo, Banyuwangi, Bojonegoro) in East Java, Indonesia
Data accessibility	Data included in this article

## Value of the data

• The dataset can be used to understand strategies for improving nursing services, especially related to the practice of inter-professional collaboration for nurses in hospitals.
• This dataset is valuable for researchers who are interested not only in mapping interprofessional collaborative practice in caring for patients, but also in analyzing and predicting interprofessional education practice models in education and clinics.
• The data emphasize the importance of developing innovative approaches to nursing services in hospitals.
• Nurse's perception of interprofessional collaboration is important and beneficial to nurses' interests in relationships and building and implementing interprofessional collaboration.
• Interprofessional collaboration practices are very useful for determining interprofessional collaboration practice models in hospitals, especially Islamic hospitals

## 1 Data description

Table 1 shows the socio-demographic characteristics of the nurses. The total number of respondents was 312. The median age of nurses was 32.55 ± 8.39 years (ranging from 20 to 50 years). In term of level of education, 183 nurses (58.65%) have Diplomas, 121 nurses (38.78%) have Undergraduate degrees, and 8 nurses (2.56%) have Masters degrees.

**Table 1:** Socio demographic characteristics of nurses at selected Muhammadiyah hospitals, Surabaya, 2019.

Characteristics of	Frequency	Percentage
Sex		
Male	81	25.96
Female	231	74.04
Age	Mean = 32.55	SD = ±8.39
20–30 years	145	46.47
31–40 years	103	33.01
41–50 years	64	20.51
Level of Education		
Diploma degree	183	58.65
Bachelor degree	121	38.78
Master degree	8	2.564
Working Experience		
Less than 5 years	105	33.65
5–10 years	92	29.49
11–15 years	41	13.14
16–20 years	40	12.82
> 20 Years	34	10.9
Ward		
Medical	199	63.78
Surgical	33	10.58
Emergency	33	10.58
Intensive Care Unit	20	6.41
Pediatric	10	3.205
Neonatal	14	4.487
Maternity	3	0.962

Table 2 shows the inter-professional collaboration perception score that averaged 97.5 ± 8.59 (ranging from 46 to 120). Data obtained through inter-professional collaboration questionnaire on nurses' perception consisted of 24 items

**Table 2:** Distribution of nurses' perception toward inter-professional collaboration between of nurses at selected Muhammadiyah hospitals, Surabaya, 2019.

No	Question	SD (%)	D (%)	U (%)	A (%)	SA (%)
1	Inter-professional collaboration is only done for certain patients	60 (19.2)	145 (46.5)	16 (5.1)	68 (21.8)	23 (7.4)
2	Nurses should solve their own problems in the event of disagreement about resolving problems related to patient care.	57 (18.3)	178 (57.1)	20 (6.4)	44 (14.1)	13 (4.2)
3	The nurse should jointly consider a friend / colleague's proposal about their patient's continued treatment plan.	3 (0.96)	1 (0.32)	5 (1.6)	136 (43.6)	167 (54)
4	Nurses should share information to verify the effects of treatment on patients in the hospitals.	1 (0.32)	0 (0.0)	3 (1.0)	168 (53.8)	140 (45.0)
5	Nurses should have a different understanding of treatment plans for patients in the hospitals.	21 (6.73)	131 (42.0)	49 (16.0)	78 (25.0)	33 (11.0)
6	Nurses should understand the reasons behind changing treatment plans for their patients with follow-up plans.	3 (0.96)	2 (0.64)	10 (3.2)	205 (65.7)	92 (29.0)
7	Nurses should check whether their patients are showing any signs of side effects or complications.	5(1.6)	12 (3.85)	13 (4.2)	187 (59.9)	95 (30.0)
8	Nurses should share information about their patients’ condition / status of their illness and the method of treatment with patients.	3 (0.96)	9 (2.88)	15 (4.8)	159 (51.0)	126 (40.0)
9	Nurses should share patient information with their patients on how best to go about with their daily life	2 (0.64)	9 (2.88)	19 (6.1)	187 (59.9)	95 (30.0)
10	Nurses should provide information on the condition of their patients even if not asked	11 (3.53)	44 (14.1 )	33 (11.0)	144 (46.2)	80 (26.0)
11	Nurses should understand all instructions given by doctors as per documented in their patients’ medical record	2 (0.64)	7 (2.24)	12 (3.8)	156 (50.0)	135 (43.0)
12	Nurses should discuss with their patients about their conditions	2 (0.64)	33 (10.6)	49 (16.0)	171 (54.8)	57 (18.0)
13	Nurses should help each other nurses in helping all the patients being treated	1 (0.32)	7 (2.24)	5 (1.6)	169 (54.2)	130 (42.0)
14	Nurses should help solve problems facing all their patients with their agreement	1 (0.32)	0(0.0)	6 (1.9)	143 (45.8)	162 (52.0)
15	Nurses should value the contribution and discussion with other colleagues about patients being treated at the hospital.	3 (0.96)	7 (2.24)	7 (2.2)	161 (51.6)	134 (43.0)
16	Nurses should share assignments with other colleagues in attending to patients in the hospital.	1 (0.32)	2 (0.64)	5 (1.6)	181 (58.0)	123 (39.0)
17	Nurses should have group discussion pertaining to patient care.	1 (0.32)	4 (1.28)	17 (5.4)	179 (57.4)	111 (36.0)
18	Nurses should share tasks among themselves in taking care of patients.	2 (0.64)	0(0.0)	7 (2.2)	188 (60.3)	115 (37.0)
19	Nurses should review all their patients’ overall health status systematically.	1 (0.32)	6 (1.92)	6 (1.9)	142 (45.5)	157 (50.0)
20	Nurses should report to doctors if any of their patients’ blood pressure are within these range: systolic blood pressure 〈90 mmHg /〉 130 mmHg or blood pressure diastolic> 90 mmHg / <60 mmHg.	4 (1.28)	20 (6.41)	29 (9.3)	170 (54.5)	89 (29.0)
21	Nurses should report to doctors if any of their patients’ temperature are within these range: <36 °Celsius or ≥37.5 °Celsius,	6 (1.92)	52 (16.7)	29 (9.3)	158 (50.6)	67(21.0)
22	Nurses should report to doctor if any of their patients’ pulse are within these range: 〈60x / minute or〉 100x / minute.	2 (0.64)	27 (8.65)	19 (6.1)	186 (59.6)	78 (25.0)
23	Nurses should report to doctors if any of their patients’ respiratory condition is > 24x / minute.	0(0.0)	45 (14.4)	22(7.1)	170 (54.5)	75 (24.0)
24	Nurses should act professionally similar to doctors when interacting with, taking care of and providing treatment to patients.	0(0.0)	13 (4.17)	11 (3.5)	125 (40.1)	163 (52.0)

Score Mean = 97.5, SD = ± 8.59, Minimum score = 46, Maximum score = 120.

SD: Strongly disagree, D: Disagree, U: Uncertain, A: Agree, SA: Strongly agree.

Table 3 shows the inter-professional collaboration practices score that averaged 39.3 ± 1.49 (ranging from 28 to 41). Data obtained through inter-professional collaborative practices questionnaire had 5 items questions

**Table 3:** Nurses’ responses toward inter-professional collaborative practices at selected Muhammadiyah hospitals, Surabaya, 2019.

No	Question	Multiple responses	Yes(Y) (%)	No (N) (%)
1	Inter-professional collaborative practices are rendered to?	Special patients (certain)	173 (55.4)	139 (44.6)
		All patients	261 (83.7)	51 (16.3)
2	A nurse will try to resolve problem involving care for a patient ….?	Individually	66 (21.2)	246 (78.8)
		Together with another nurse	307 (98.4)	5 (1.6)
		Together with the doctor	298 (95.5)	14 (4.5)
3	Nurses practice inter-professional collaboration to take care of their patients in these manner:	Verifying the effects of treatment when facing patient treatment in hospital	299 (95.8)	13 (4.2)
		Advanced care plans	0 (0.0)	312 (100.0)
		Changes in treatment plans	296 (94.9)	16 (5.1)
		Physical examination, side effects or complications of the patient	302 (96.8)	10 (3.2)
		Treatment methods	293 (93.9)	19 (6.1)
		Sharing information on the level of independence and ADL of patients	302 (96.8)	10 (3.2)
4	Nurses practice inter-professional collaboration through the following:	Joint participation	302 (96.8)	10 (3.2)
		Information sharing	305 (97.8)	7 (2.2)
		Negotiation	263 (84.3)	49 (15.7)
		Mutual assistance	305 (97.8)	7 (2.2)
		Solve the problem	311 (99.7)	1 (0.3)
5	Nurses cooperate with each other by:	Helping each other	305 (97.8)	7 (2.2)
		Participate in problem-solving related to their patients	300 (96.2)	12 (3.9)
		Appreciating the contribution of each member	296 (94.9)	16 (5.1)
		Sharing assignments	302 (96.8)	10 (3.2)
		Carry on the tasks for which they are responsible	304 (97.4)	8 (2.6)

Score Mean = 39.3, SD = ± 1.49, Minimum score = 28, Maximum score = 41.

Nurses’ perception of inter-professional collaboration is measured using a Likert scale measure. In term of perception, 146 respondents (46.8%) had negative perceptions and 166 nurses had positive perceptions (53.2%) (see Table 4). Whereas, in terms of inter-professional collaborative practices, 157 respondents (50.4%) had good collaborative practices and 155 nurses had poor collaborative practices (49.6%).

Table 4 portrays that the highest nurses' perception towards inter-professional collaboration is nurses who have work experience of more than 20 years (70.6%) and nurses who have practical ability of proficient inter-professional collaboration (55,9%)

## 2 Experimental design, materials, and methods

A cross-sectional study was conducted involving nurses at Muhammadiyah hospitals in East Java province, from June 2019 to December 2019. Nurses who worked in inpatient and outpatient rooms were chosen as respondents, those with a minimum of one year work experience. The sample size came to 312 nurses according to similar studies [12,13]. Samples were selected by random sampling [14]. A self-administered research questionnaire was used for data collection. The inter-professional collaboration questionnaire on nurses' perception consisted of 24 items measured using a 5-point Likert scale [1], [2], [3], [4], [5], [6], [7], [8], [9], [10], [11]. The scores for the scale ranged as follows: 1 (Strongly Disagree), 2 (Disagree), 3 (Uncertain), 4 (Agree) 5 (Strongly Agree). Cronbach's alpha for the nurses‘ perception variable was 0.91. The inter-professional collaborative practices questionnaire had 5 items questions with multiple responses Yes (Score 1), No (Score 0) answers. Cronbach's alpha for the Practice of Inter-professional Collaboration variable was 0.97 [3,15]. The descriptive data analysis in the form of a percentage was performed using SPSS Statistics software for Windows version 16 (IBM Corp., Armonk, NY). The research was approved by the Ethics Committee of the Muhammadiyah University of Surabaya (Ethical approval number: 014.0703.19). Verbal consent was obtained from each participant, and the anonymity and confidentiality of the participants were maintained.

## Declaration of Competing Interest

The author declares that he has no financial interests or personal relationships that could have appeared to affect the work reported in this paper.
